# A randomized controlled trial for prevention of acute exacerbation of stable chronic obstructive pulmonary disease with acupoint application of traditional Chinese medicine: Study protocol clinical trial (SPIRIT Compliant)

**DOI:** 10.1097/MD.0000000000019396

**Published:** 2020-03-06

**Authors:** Chuantao Zhang, Hongjing Yang, Wenfan Gan, Jun Chen, Yang Yang, Wei Xiao, Kunlan Long, Keling Chen, Qingsong Huang, Peiyang Gao

**Affiliations:** a Department of Respiratory Medicine; b Department of Critical Care Medicine, Hospital of Chengdu University of Traditional Chinese Medicine, Chengdu, PR China.

**Keywords:** acupoint application, acute exacerbation, randomized controlled trial, stable COPD, traditional Chinese medicine

## Abstract

**Introduction:**

Chronic obstructive pulmonary disease (COPD) is a major public health problem that severely affects the quality of life of patients and may even endanger their lives. Although modern medicine has achieved significant results in relieving the clinical manifestations of COPD, it is difficult to prevent its progression and acute exacerbation entirely. As one of the classic aspects of acupuncture and moxibustion therapy, acupoint application of traditional Chinese medicine (TCM) can improve the clinical efficacy of western medicine in treating COPD. To date, however,there is no high-quality clinical trial to assess the effectiveness of TCM acupoint application directly in preventing acute exacerbation of stable COPD.

**Methods:**

The study is a randomized, double-blind, placebo-controlled trial, in which 200 stable COPD patients will be randomly and equally divided into the experimental group or control group. Both groups will undergo standard Western medicine treatment; however, the patients in the experimental group will be also treated with TCM acupoint application, while the control group will be given placebo acupoint application. The duration of the treatment will be 1 month and a follow-up for 11 months. The primary outcome will be the number of acute exacerbation episodes of COPD, and the secondary outcomes will include the lung function, St George's Respiratory Questionnaire, COPD Assessment Test, and 6-Minute Walk Test. A safety assessment will also be performed during the trial.

**Discussion:**

The aim of this study is to evaluate the efficacy and safety of TCM acupoint application in preventing acute exacerbation of stable COPD. Our study will provide sound evidence to support the evidence-based medicine of TCM acupoint application as an additional measure in the prevention of acute exacerbation of stable COPD.

**Trial registration:**

ChiCTR1900026564, Registered 14 October, 2019

## 1 Introduction

Chronic obstructive pulmonary disease (COPD) is one of the leading causes of morbidity and mortality in the world.^[1]^ It is estimated that 384 million people worldwide suffer from COPD,^[2]^ of which China accounts for 100 million.^[3]^ In 2017, 3.2 million people died of COPD, a toll that is expected to reach 4.4 million per year by 2040.^[1]^ Acute exacerbation episodes of COPD occur 0.5 to 3.5 times a year, which lead to further deterioration of airway injury, promotes the progression of the disease, and causes a huge economic burden on patients.^[2–4]^ Therefore, the key to COPD treatment is to treat it effectively during its stable period to prevent the occurrence of acute exacerbation.

Inhaled bronchodilators in stable COPD are central to symptom management. If necessary, triple therapy (inhaled glucocorticoids + long-acting β2-agonist + long-acting muscarinic receptor antagonist) may be considered to reduce the deterioration frequency and increase labor tolerance. However, it cannot be confirmed if these drugs completely prevent disease progression and control COPD at different stages.^[5]^ Moreover, these drugs have certain adverse reactions, such as palpitations, arrhythmia, and tremor, which limit the application of the drugs to a certain extent.

Acupuncture has been confirmed by some meta-analyses and clinical studies to safely and effectively treat patients with COPD and improve the exercise capacity as per the 6-Minute Walk Test (6MWT) and the quality of life of COPD patients as per the St George's respiratory questionnaire (SGRQ).^[6–7]^ Acupoint application therapy is a compound treatment method that integrates meridians, acupuncture points, and herbs. First, the processed herbal ointment is placed on the medical adhesive medicine plaster. Then, some points are affixed with medicinal paste that has the characteristics of being non-traumatic, needing only small dose of medication, and having a direct effect on the acupuncture points. According to the basic TCM theory, acupuncture points application is similar to acupuncture in regulating meridians, yin and yang, and qi as well as the blood of the human body. Chinese medicines commonly used in acupoint application include Bai Jiezi (Sinapis Semen), Ma Huang (Ephedrae Herba), Xi Xin (Asari Radix Et Rhizoma), Gan Sui (Kansui Radix), Yan Husuo (Corydalis Rhizoma), and Sheng Jiang (Zingiberis Rhizoma Recens). A meta-analysis based on Chinese literature revealed that Bai Jiezi, Xi Xin, Gan Sui, and Yan Husuo, which were applied to acupoints (Feishu [BL 13], Dazhui [GV 14], and Danzhong [BL 43]) were considered as the basic medicines. Furthermore, those acupoints were considered as the basic acupoints to treat chronic respiratory diseases, such as asthma, COPD, chronic bronchitis, and allergic rhinitis. The therapeutic effect could be related to the anti-inflammatory effects of Bai Jiezi and Gan Sui.^[8–9]^

Acupoint application has been recorded in the Compendium of Materia Medica in detail. It is a common therapy for chronic pulmonary diseases, such as COPD, and it has been used by Chinese doctors for about 1300 years. Since the 1950s, acupoint application therapy has been widely carried out in 30 provinces, municipalities, and autonomous regions in China. A meta-analysis based on Chinese clinical trials (3481 out of 32 randomized control trials [RCTs]) has shown that different combinations of TCM and acupoint application can improve the clinical efficacy of Western medicine in treating COPD.^[10]^ To date, however, there is no high-quality clinical trial to assess the effectiveness of Chinese medicine acupoint application directly in preventing acute exacerbation of stable COPD. Therefore, we designed this clinical trial. To the best of our knowledge, this study is a rare, randomized, placebo-controlled design of RCT to evaluate the efficacy and safety of acupoint application of Chinese medicine in preventing the acute exacerbation of stable COPD.

## 2 Methods/design

### 2.1 Design

**Figure 1:**
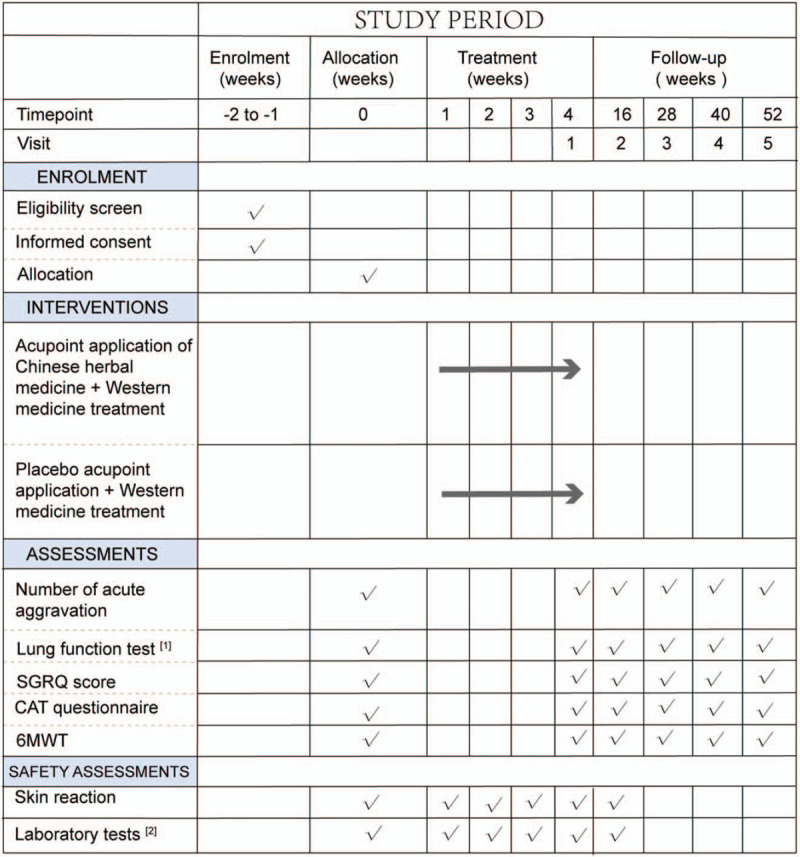
SPIRIT figure: Schedule of enrollments, interventions, and assessments. [1] Lung function tests: FEV_1_, forced expiratory volume in one second; FVC, forced volume capacity. [2] Laboratory tests: blood, urine, feces, electrocardiogram, and kidney and liver function tests. 6MWT = 6-minute walk test, CAT = COPD assessment test, SGRQ = St George's Respiratory Questionnaire.

**Figure 2:**
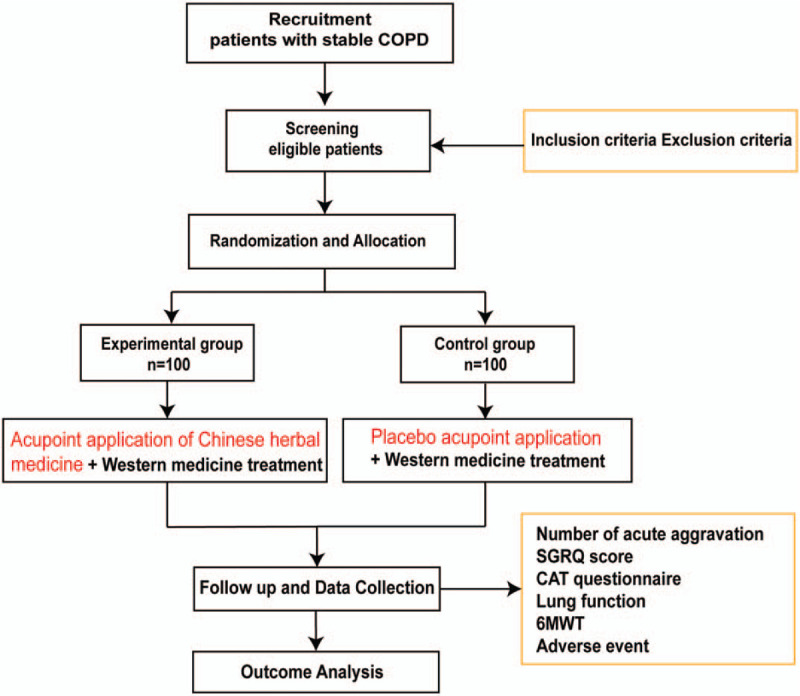
Illustration of the design for clinical studies. 6MWT = 6-minute walk test, CAT = COPD assessment test, COPD = chronic obstructive pulmonary disease, SGRQ = St George's Respiratory Questionnaire.

This study is a prospective, double-blind, randomized, placebo-controlled clinical trial. To prevent design bias, we will follow the Consolidated Standards of Reporting Trials statement^[11]^ and the Standard Protocol Items: Recommendations for Interventional Trials (SPIRIT) 2013 statement.^[12]^ This trial will include 200 participants, and the participants will be informed of the benefits and risks of the study in detail. After obtaining written informed consent, eligible participants will be randomly assigned to the experimental group and control group in a ratio of 1:1. The entire study includes a screening assessment period, a 2-week run-in period, a 1-month treatment period, and an 11-month follow-up period. The schedule of enrollments, interventions, and assessments is shown in Figure 1 (the SPIRIT figure). The illustration of the design for clinical studies is presented below in Figure 2. The SPIRIT 2013 checklist is presented in Additional File 1.

### 2.2 Ethics approval

The study is in compliance with the *Declaration of Helsinki* (Edinburgh 2000 version). The research program has been approved by the China Ethics Review Committee for Registered Clinical Trials (Approval No. ChiECRCT20190221), and the members of the ethics committee tracked the design and implementation of the research. We registered the study in the Chinese Clinical Trial Registry (Registration No. ChiCTR1900026564) in 2019. If there is any amendment to the protocol, approval must be again sought from the Ethics Committee.

### 2.3 Recruitment

This study will be conducted in the Hospital of Chengdu University of TCM (Chengdu, China). Participants will be recruited through a recommendation by the respiratory clinician and social media. Before enrollment, participants will be provided with detailed information about the clinical study, including its purpose, processing, scheduling, and possible risks and benefits. Only those who agree to sign the informed consent and voluntary participation in the trial will be included in the study.

### 2.4 Sample size

Sample size calculations are based on the primary outcome (the number of acute attacks of COPD within a year). According to the previous literature,^[13]^ it is estimated that the number of acute exacerbation episodes of COPD in the control group is 2.29, with a standard deviation of about 1.23. After acupoint application of Chinese herbal medicine (CHM), the number of attacks in the experimental group is 1.64 times. At the 5% significance level, a total of 76 patients per group is required to achieve 80% power. The software Power Analysis and Sample Size version 11.0 (PASS 11.0) was used to calculate the sample sizes of the experimental group and the control group as 76 cases each. With an estimated dropout rate of 20%, a total of 190 patients is enrolled. In the actual study, 100 cases will be included in each group.

### 2.5 Randomization and allocation concealment

A statistician, the member of the Sichuan TCM evidence-based Medicine Center will generate 200 random serial numbers using the SAS 9.2 Software (SAS. Cary, USA), and stratified randomization will be based on the GOLD classification of COPD. Eligible patients will be randomized into the experimental or control groups in a 1:1 ratio. The group numbers will be provided in a continuous manner in sealed envelopes made from carbonless paper. The envelopes will be kept by a study administrator who will not directly participate in the recruitment or follow-up of any participant. The administrator will open one envelope and provide the participant with their group number on the day of inclusion. Until the completion of the trial, the participants, the clinical practitioners, the outcome evaluators, the data manager, and statistician will not know the treatment allocations.

### 2.6 Blinding

This trial is a double-blind design in which neither the acupoint application therapists nor the participants will be aware of their treatment group during the trial period. Both placebo paste and CHM paste will be produced, packaged, and marked by Sichuan Green Pharmaceutical Technology Development Co., Ltd. to ensure that they are consistent in shape, smell, and color. In addition, the research team will be instructed not to communicate with the participants regarding their possible treatment group allocation. Only in emergencies, such as serious adverse events, or if the patient needs emergency treatment, can the researchers report to the principal researcher to decide whether to expose the blind.

### 2.7 Diagnostic criteria

All participants must meet the Western medicine diagnostic criteria for COPD (Table 1)^[14]^ and the TCM syndrome diagnostic criteria of *lung-spleen qi deficiency syndrome* (Table 2).^[15]^ The determination of syndrome differentiation shall be determined independently by two designated deputy physicians of TCM.

**Table 1:**
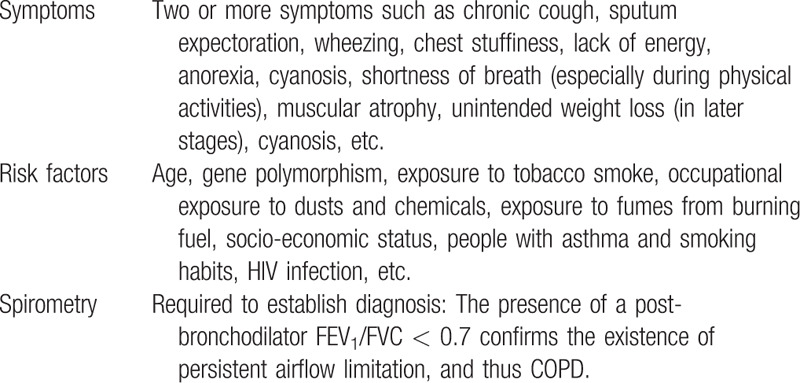
Western medicine diagnostic criteria for chronic obstructive pulmonary disease^[14]^.

**Table 2:**
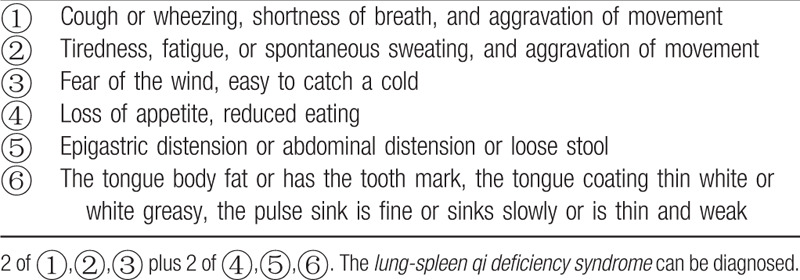
Diagnostic criteria for Traditional Chinese Medicine differentiation of *lung-spleen qi deficiency syndrome*^[15]^.

### 2.8 Eligibility criteria

Inclusion criteria:

(1) Age range 45 to 70 years, male or female
(2) Grade 2–3 of the COPD diagnostic criteria in the Global Initiative for Chronic Obstructive Lung Disease (GOLD) guidelines:^[14]^ 20 minutes after inhalation of salbutamol 400 μg, forced expiratory volume in 1 second/forced vital capacity (FEV_1_/FVC) <70% and 30% predicted value ≤FEV_1_<80% predicted value
(3) Patient is in a stable phase of COPD with no respiratory infection and acute exacerbation of COPD in the past 4 weeks
(4) Patients with lung-spleen qi deficiency syndrome that meets the diagnostic criteria for common syndrome of TCM
(5) Participants volunteer to participate in the research and provide informed written consent

Exclusion criteria:

(1) Patients with a combination of chronic lung diseases, such as bronchial asthma, interstitial lung disease, active tuberculosis, or bronchiectasis, requiring intervention or treatment
(2) Patients with a combination of severe primary diseases, such as severe mental disorders, cardiovascular and cerebrovascular diseases, liver and kidney diseases, endocrine diseases, hematopoietic system diseases, malignant tumors, etc
(3) Presence of skin wounds, skin ulcers, and skin infections on the application site
(4) Patients allergic to CHM, excipients, or dressings used in this project
(5) Patients with limited limb movements
(6) Women who are breastfeeding, pregnant, or preparing for pregnancy
(7) Patients participated in any other clinical studies in the past 6 months.

### 2.9 Termination and withdrawal criteria

Participants will be informed that they have the right to discontinue treatment and withdraw from the research project for any reason at any time, and the reason for withdrawal will be recorded in the CRF. If they quit, they will receive standardized treatment. The criteria for stopping treatment and withdrawal from research projects are as follows:

(1) There is severe allergy to the research dressings and drugs during the treatment, and the patient cannot continue being a part of the clinical research.
(2) Participant's compliance is poor, or other drugs prohibited by this research scheme are being consumed.
(3) Voluntary withdrawal from the trial or loss of follow-up.

## 3 Interventions

### 3.1 Treatment plan

**Figure 3:**
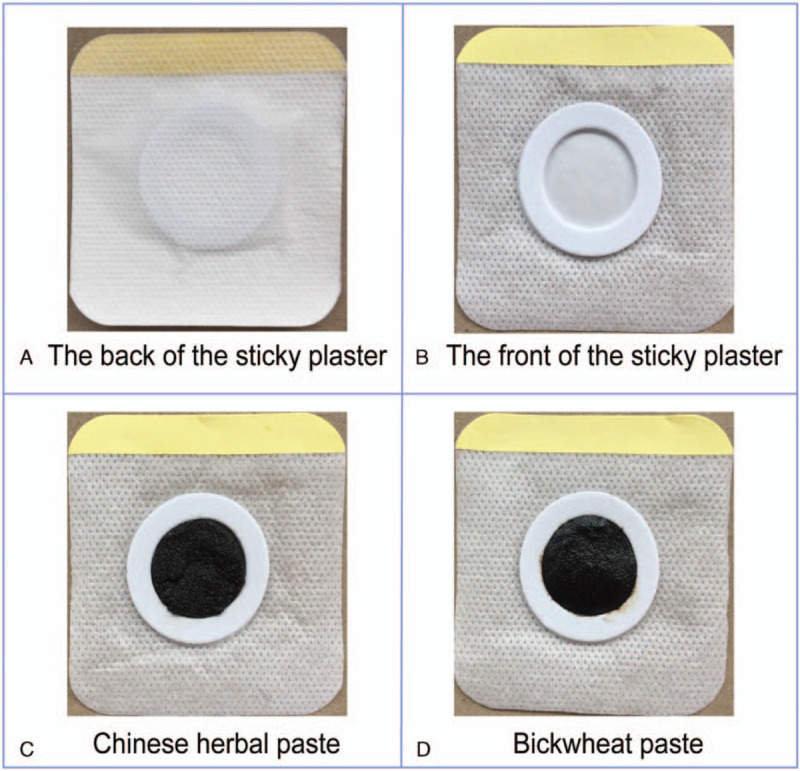
Illustration of the sticky plaster. (A) appearance of the acupoint plaster from the front; (B) appearance of the acupoint plaster from the back; (C) Chinese herbal paste on the plaster; (D) Buckwheat plaster; both (C) and (D) are identical in appearance.

Both groups will be treated with standard Western medicine. The treatment plan is based on the GOLD^[14]^ and the Guidelines for the Diagnosis and Treatment of Chronic Obstructive Pulmonary Disease (Revised 2013).^[16]^ All participants will be taught to use inhaled agents correctly, and they would have to quit smoking and prevent catching colds. Drug selection (based on the patient's pulmonary function) and symptoms and risk of acute exacerbation will be stratified, including that of GOLD grade 2 - regular inhalation of tiotropium bromide powder for inhalation (Si Li Hua, Boehringer Ingelheim Pharma GmbH & amp; Co.KG [Germany], 18 μg × 30 capsules), 18 μg per inhalation, once a day; and GOLD grade 3 - regular use of budesonide and formoterol fumarate powder for inhalation (Symbicort Turbuhaler, AstraZeneca AB [Sweden], 160 μg/4.5 μg/inhalation, 60 inhalations), 164.5 μg per inhalation, twice a day. The experimental group will be treated with acupoint application of CHM at the same time, while the control group will be treated with acupoint application of placebo. Acupoint plaster treatment will be performed by certified acupuncturists with at least 5 years’ clinical experience (Fig. 3A and B). The course of treatment is 1 month, and the course of follow-up will be once every 3 months for 11 months. The TCM and acupoint selection program is based on academic literature data^[17–18]^ and the “Guidelines for the Clinical Application of Acupoint Application of Winter Diseases and Acupuncture Points (Draft)”.^[19]^ The placebo used in the control group is made of buckwheat flour without any herbs, but has added honey and ginger juice to maintain an appearance similar to that used in the experimental group (Fig. 3C and D), thus making the plaster used in the 2 groups indistinguishable for patients and therapists. The standard operating procedure (SOP) for acupoint application is as follows:

(1) Prepare the herbal materials: Bai Jiezi (Sinapis Semen), Ma Huang (Ephedrae Herba), Huang Qi(Astragali Radix), Ban Xia (Pinelliae Rhizoma), Xi Xin (Asari Radix Et Rhizoma), and Gan Sui (Kansui Radix) are mixed in a dose ratio of 3:3:2:1:1:2. The raw materials are ground into fine powder (diameter <75 μm) by a special powder mixer of CHM.
(2) Add the liquid base: Put the mixed herbal powder into the container, add ginger juice and honey (1:2), and mix well to make a thick herbal ointment.
(3) Add the sticky plaster: Take 10 g ointment and place it evenly on the center of the specially made medical sticky plaster.
(4) Places of application: Feishu (BL13, bilateral), Dingchuan (EX-B1, bilateral), Gaohuang (BL43, bilateral), Zusanli (St36, bilateral), Dazhui (GV14), and Danzhong (CV17)
(5) Treatment procedures: To find the acupuncture points, first disinfect the application site with iodophor routinely, and then stick the plaster on the acupuncture points. The time of each application is 3 to 4 hours, depending on the individual reaction of each patient. We considered the patient's personal constitution and tolerance, and usually the patient can tolerate the plaster. If patients consciously presented any discomfort, such as obvious burning sensation, severe itching, or blisters, they would be advised to immediately remove the ointment application and deal with it accordingly. The treatment will be performed twice a week for 4 weeks, total 8 times.

### 3.2 Outcome measures

*Primary outcome*: The primary outcome includes the number of acute exacerbation episodes of COPD (at week 52). COPD exacerbation is defined as an acute worsening of respiratory symptoms resulting in additional therapy.^[20]^

*Secondary outcomes*: at weeks 4, 16, 28, 40, and 52

(1) FEV_1_ and FEV_1_/FVC change values of pulmonary function measurement.
(2) Changes in the SGRQ score
(3) Changes of the COPD Assessment Test score
(4) 6MWT

### 3.3 Safety assessment

The dosage of CHM used in this study is within the recommended range of the Pharmacopoeia of the People's Republic of China (2015 edition), and all operating procedures will strictly follow the SOP. In this study, patients could be allergic to research drugs or dressings during the application period, such as rash, skin itching, skin redness and swelling, or skin burning. We will strictly adhere to the criteria and avoid including those who are allergic to TCM or the accessories/dressings used in this study. Moreover, laboratory tests will be performed to analyze blood, urine, feces, electrocardiogram, and kidneys and liver function, from the time of enrollment through the treatment period.

### 3.4 Compliance

In this study, certain measures will be taken to ensure the compliance of the participants. Before entering the group, the participants will be carefully screened to understand their history of previous drug use, detailed information on the test content, course of treatment, adverse reactions of the treatment, and some possible burdens on the participants. Furthermore, all examination and transportation costs will be covered and the results of physical examinations will be explained at every visit. Prior to every visit, messages will be sent through WeChat or by phone to remind patients of the upcoming data collection. In addition, ongoing support, such as free registration and treatment advice, will be provided to the participants in the follow-up phase.

### 3.5 Adverse events

All details of relevant and unexpected AEs, such as the time, severity, and suspicious causes of the AEs, will be recorded in detail in the case report files (CRF). Individuals reporting mild and moderate AEs will be treated for symptoms and closely observed. If serious AEs occur, the patients will be admitted to the hospital immediately for systematic treatment and reported to the Steering Committee and Ethics Committee within 24 hours.

### 3.6 Data management and quality control

The researchers in the trial team will be required to attend a training workshop before the trial begins. Each one will receive a copy of the trial protocol, and they will be asked to adhere to the protocol throughout the study. Data management should be carried out according to the standard operating procedure of data management, and data entry and sorting will be done using EpiData 3.1 software. Two database operators will independently enter the same data, then conduct data consistency test, and proofread it multiple times. Files are stored in a secure and accessible manner. Security measures will be managed by user identification codes and passwords, and monthly backups are stored on CD media. Finally, the database is locked and analyzed according to the protocol, and confirmed and reviewed by the main researchers. The Sichuan TCM evidence-based Medicine Center (Chengdu, China), which does not have any competing interests, will be responsible for monitoring the data. The Department of Science Research of the hospital at Chengdu University of TCM, which is independent of the investigators, will perform data audits in the middle of the trial.

### 3.7 Statistical analysis

We will use the Statistical Package for the Social Sciences version 21.0 (SPSS 21.0, Chicago, IL) statistical software for analysis, and use subgroup analysis methods to explain the efficacy and safety of acupoint sticking in different grades of GOLD patients to obtain comprehensive information. The measurement data will be examined using group *t* tests or non-parametric tests, the count data will be tested using a Chi-square test or Fisher exact probability method, and the grade data will be tested using non-parametric tests. All statistical tests will be bilateral tests and *P* values <.05 will be considered to indicate statistical significance.

A full analysis set (FAS, including completed trials, shedding cases and excluded cases) will be used for baseline data, and the FAS set and per-protocol (PP) set will be used for efficacy evaluation (PP set, including completed cases, and excluding shedding cases and rejection cases). If the two analytical conclusions are consistent, the credibility of the test results can be enhanced. If not, their differences should be fully discussed and explained. The missing data should be replaced by the method of sequence mean value in the FAS.

## 4 Discussion

COPD is the most common chronic respiratory disease. In China, COPD shows the “four high” characteristics of high morbidity, high disability rate, high mortality rate, and high disease burden, and it has become one of the most prominent public health and medical problems.^[21]^ Although modern medicine has a good effect in relieving the symptoms of COPD, these measures still cannot fully control the disease progression and prevent acute exacerbation.^[22]^ There is still a need to continue to develop new interventions to improve efficacy. CHM acupoint application is a kind of pulmonary rehabilitation method based on the theory of TCM, which applies CHM to certain acupuncture points of the human body to treat and prevent diseases. It is one of the suitable techniques of TCM, designated and popularized by the administration of TCM. It has a wide range of population usage and social influence in China. Although there have been many clinical reports on the intervention of CHM acupoint application in the treatment of COPD, there are still many problems in the design of these studies.^[23]^ The use of random methods is not rigorous, and there is no description of blinding and allocation hiding. Additionally, the correct sample calculation method was not offered, AEs were not recorded in detail, and the intention-to-treat (ITT) analysis not performed on the shedding and missing cases is prone to bias risk. Therefore, we designed this placebo-controlled RCT to evaluate the efficacy and safety of CHM acupoint application in preventing acute exacerbation of stable COPD.

Compared with previous studies, we focus on assessing the effects of acupoint application on the long-term efficacy of stable COPD (future acute exacerbation) and patient activity tolerance. However, it cannot be ignored that this study also has certain limitations. First, in clinical practice, pure COPD patients are rare, and most patients present with other diseases such as cor pulmonale, hypertension, diabetes, and coronary heart disease. Therefore, the practicability (extrapolation) of the results in this study is not clear and needs to be further evaluated. Second, the study is being performed in Sichuan, China, and it is uncertain whether the relative effects of the trial drugs would be similar in other ethnic groups. Despite the limitations, we believe that this study will be helpful in finding the long-term benefits of CHM acupoint application in the prevention of acute exacerbation of stable COPD. In the future, multi-center RCTs should be conducted and multi-dimensional comparison should be conducted.

## 5 Others

### 5.1 Trial status

This paper is based on protocol version 2.0 dated 3 November 2019. The clinical study began from Dec, 2019, and approximate date of completion is December 2021. Currently, participant recruitment is ongoing.

## Acknowledgments

We are grateful to the Sichuan Science and Technology Program for funding this study. We also would like to thank Editage (www.editage.cn) for English language editing.

## Author contributions

**Conceptualization**: Chuantao Zhang, Qingsong Huang.

**Investigation**: Jun Chen, Wenfan Gan.

Qingsong Huang orcid: 0000-0003-1878-3579.

**Supervision**: Peiyang Gao, Wei Xiao.

**Writing – original draft**: Hongjing Yang, Yang Yang.

**Writing – review & editing**: Kunlan Long, Keling Chen.
